# The Effect of Bevacizumab and Propranolol on Nasal Polyposis

**DOI:** 10.1155/2022/6174664

**Published:** 2022-10-12

**Authors:** Yuksel Olgun, Safiye Aktas, Semih Sutay, Mustafa Cenk Ecevit

**Affiliations:** ^1^Dokuz Eylül University School of Medicine, Department of Otorhinolaryngology, Izmir, Turkey; ^2^Dokuz Eylül University Institute of Oncology, Department of Basic Oncology, Izmir, Turkey

## Abstract

**Objective:**

This study aims to evaluate the effects of bevacizumab and propranolol from the point of view of a possible antiangiogenic effect in a model of primary nasal polyp (NP) tissue culture.

**Methods:**

NP samples of 21 patients and normal healthy nasal mucosa samples of 7 patients were cultured. Samples were divided into four groups as follows (healthy nasal mucosa, NP without any treatment, NP treated with propranolol, NP treated with bevacizumab). Cultured tissues were formalin fixed and paraffin embedded. Tissue sections and immunohistochemical VEGF-A, angiopoietin-1 (Ang-1), and angiopoietin-2 (Ang-2) expressions were evaluated. ELISA was also performed for each one of them.

**Results:**

Both propranolol and bevacizumab significantly decreased the expressions of VEGF-A and Ang-1, and they significantly increased the expression of Ang-2 in comparison to the control NP group. In the healthy nasal mucosa group, no significant expression of VEGF-A was seen, a slight (+) Ang-1 expression, and a high (+++) Ang-2 expression were observed.

**Conclusion:**

Bevacizumab and propranolol exert an antiangiogenic effect on NP tissues, mainly by decreasing VEGF-A and Ang-1 expression, increasing Ang-2 expression.

## 1. Introduction

Nasal polyposis is a chronic inflammatory disease of the sinonasal mucosa. Chronic rhinosinusitis with nasal polyposis (CRSwNP) is defined as bilateral, endoscopically visualized polyps in the middle meatus [1]. As a consequence of nasal mucosal inflammation, a cascade of events results in prolapsed nasal mucosa, which manifest as nasal polyps [1, 2]. In clinical practice, nasal polyps represent nasal obstruction, smell and taste disorders, headache, and nasal discharge [1].

Although corticotherapy is the primary solution for NP treatment, functional endoscopic sinus surgery is needed for cases that do not respond to medical treatment [2]. Even in the best hands, revision surgery may be required in 15–20% of the cases in long-term follow-up periods [3]. For this reason, possible therapeutic effects of different biological agents like methotrexate, omalizumab, reslizumab, and mepolizubab are still investigating [4–6].

Angiogenesis is described as the formation of new blood vessels by proliferation and migration of preexisting capillaries [7]. According to recent studies, angiogenic factors and chemokines are elevated in NP tissues compared with normal nasal mucosa [5, 7–9]. However, in the literature, there are very few studies which aim to investigate the block of this increased angiogenic process in NP [5, 10].

Bevacizumab is FDA approved drug and monoclonal antibody which inhibits the angiogenesis by blocking vascular endothelial growth factor-A (VEGF-A) [11]. Although propranolol is a beta blocker drug which is used to treat cardiac arrhythmia and hypertension in recent years, it has been commonly used in the treatment of pediatric age hemangiomas treatment due to its antiangiogenic effects by potentially decreasing VEGF levels [12, 13].

The aim of our study is to evaluate the possible antiangiogenic effects of bevacizumab and propranolol in a model of primary nasal polyp culture.

## 2. Materials and Methods

This study was approved by Dokuz Eylul University noninvasive research ethical committee (2015/15–28). Nasal polyp samples of 21 patients who did not use any systemic or topical steroids and normal healthy nasal mucosa samples of 7 patients without NP were used for the study. Informed consent was taken from all patients. All 28 patients did not have a history of oral or topical usage of steroids prior twomonths of their nasal surgery. Healthy nasal mucosa samples were taken from the patients who had turbinoplasty operations for non-allergic reasons and who did not have any allergic symptoms.

Primary nasal polyp cultures were formed by using nasal polyps samples and normal healthy nasal mucosa were cultured as well. Samples were divided into four groups and VEGF-A, angiopoietin-1 (Ang-1), angiopoietin-2 (Ang-2) expressions were evaluated for each group.

### 2.1. Cell Culture Group

  Group 1 (n:7): Healthy nasal mucosa (Control group)
  Group 2 (n:7): Nasal polyposis group without any treatment
  Group 3 (n:7): Nasal polyposis group treated with 50 *μ*M propranolol
  Group 4 (n:7): Nasal polyposis group treated with 1 : 25 mg/ml bevacizumab

### 2.2. Sample Preparation

2 cm^3^ polyp and control nasal mucosa tissues were transferred to the laboratory in a 7 cc transfer medium (DMEM+ 1% penicillin-streptomycin with 1% Ambisome) in sterile conditions. They were frozen to −80° slowly in 48 hours and kept frozen in freezing medium (complete DMEM + 5% DMSO) until the test procedure.

Tissues were cut into 2 mm^3^ fragments in sterile conditions. They were incubated in complete medium. Four fragments per well were put in a 24 well plates attached to a 1 × 1 cm gelatin sponge (Spongostan, Johnson &Johnson, San Angelo, TX, USA) as an upward positions. Each condition was represented by 8 fragments.

### 2.3. Application of Drugs

The plates were shaken on a plate shaker placed in a 5% CO^2^ incubator at 37°C, with 5 RPM speed for 24 hours. The conditions were control (healthy nasal mucosa), NP without any treatment, NP and propranolol (Sigma-Aldrich, P0884) (50 *μ*M), and NP and bevacizumab (Genentech, Altuzan 100 mg/4 ml) (1.25 mg/ml).

### 2.4. Tissue Processing and Immunohistochemistry

After 24 hours incubation, tissue fragments were collected in cassettes and put in 10% formalin solution for 24 hours. After routine tissue processing and paraffin embedding, 5 *μ*m thick sections were taken on positively charged slides. Hematoxylin-Eosin stained sections were prepared for each condition and case. Immunohistochemistry was performed automatically (Ventana Discover), by a streptavidin-biotin-based method colored by the help of diaminobenzidine. Secondary antibody was antirabbit based. Polyclonal antibodies against VEGF-A (Bioss, BS4572R, USA), Ang-1 (Bioss, BS-0800R, USA), and Ang-2 (Bioss, BS-0677R, USA) were applied as primary antibodies in preoptimized 1/200 dilution. Light microscopic evaluation was carried out by a light microscope (Olympus B57). All areas of all samples in each group were assessed by microscopy. Each was recorded as separate data for statistical analysis. Diffuse expression was considered positive. Grading of the expression was done according to the intensity of brown staining.

For each antibody, immunohistochemical evaluation was done as follows; 0: negative expression, +: low expression, ++: moderate expression, and +++: high expression.

## 3. Results

### 3.1. Immunohistochemistry Results

**Figure 1:**
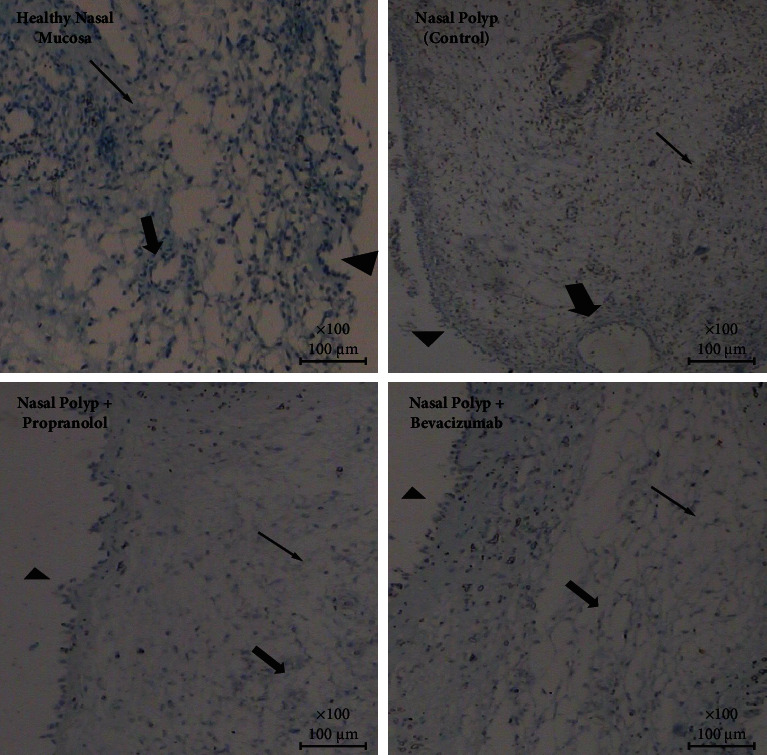
VEGF-A expressions on each group: A slight expression is observed in normal mucosa and nasal polyp group; this expression was absent in propranolol and bevacizumab group (arrow head: mucosa, thick arrow: capillaries, thin arrow: intersisium (VEGF-A, IHC, DABx40).

**Figure 2:**
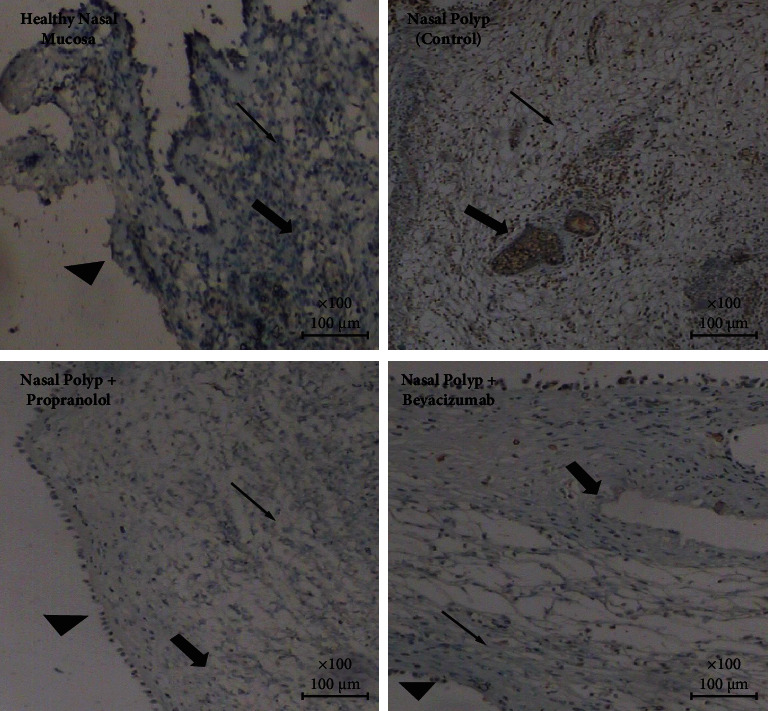
Ang-1 expressions on each group: A moderate expression is observed in nasal polyp group; this expression was absent in propranolol and bevacizumab group while it is low in normal mucosa (arrow head:mucosa, thick arrow: capillaries, thin arrow: intersisium (Ang-1, IHC, DABx40).

**Figure 3:**
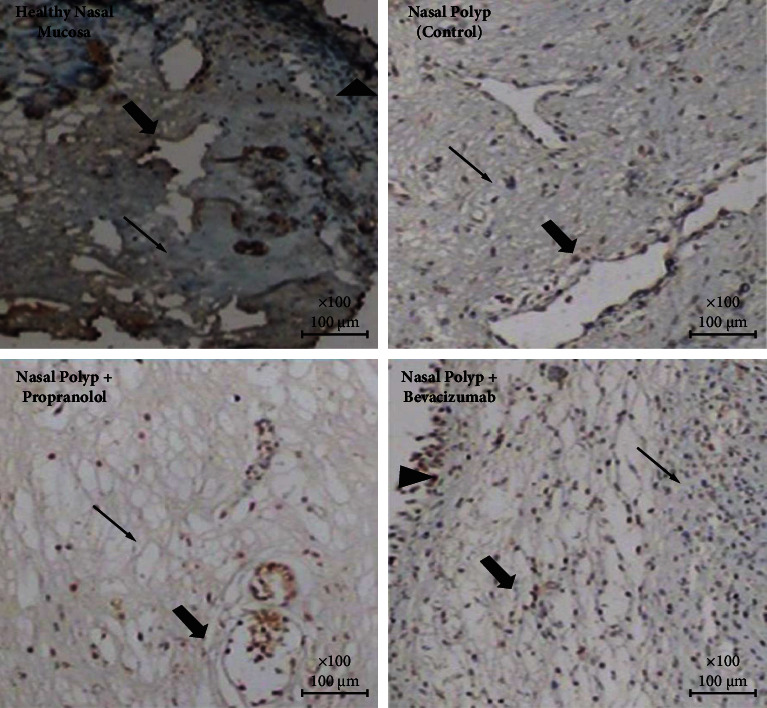
Ang-2 expressions on each group: A slight expression is observed in normal mucosa and nasal polyp group; this expression was high in propranolol and bevacizumab group (thick arrow: capillaries, thin arrow: intersisium (Ang-2, IHC, DABx40).

All of the data for polyp and normal mucosa tissues are given as a supplementary file with this paper. In group 2 (NP group), a low (+) VEGF-A and Ang-2 expression and moderate Ang-1 expression was observed. While with the application of propranolol and bevacizumab, in both treatment groups (groups 3 and 4), no significant expressions of VEGF-A (−) and Ang-1 (−) were observed but in both of these groups Ang-2 was highly (+++) expressed. In the healthy nasal mucosa group (group 1), no significant expression of VEGF-A was seen and a slight (+) Ang-1 expression and a high (+++) Ang-2 expression were observed (Table 1) (Figures 1–3).

**Figure 4:**
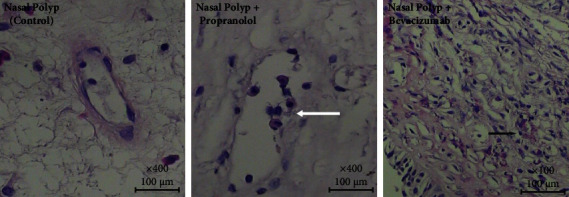
Light microscopic findings on nasal polyps groups showing endotel cell damage (thick White arrow) in Propronalol group; hyalen necrosis in Bevacizumab group (thick black arrow). (Hematoxylin-eosin stained 5 micrometer thin slides: nasal polyp control and propranolol ×400; bevacizumab ×100).

Our results have shown that with the application of both propranolol and bevacizumab separately, the expression of VEGF-A and Ang-1 decreased, while the treatment has significantly increased the expression of Ang-2. Our light microscopic findings showed that bevacizumab mainly caused hyalin necrosis in capillaries while propranolol leaded to endothelial cell damage (Figure 4).

## 4. Discussion

In our study, we found that both Ang-1 and VEGF-A expression levels were elevated in NP tissues in comparison to the control group and both propranolol and bevacizumab decreased their expression levels.

Similar studies have also shown that, in NP tissues, angiogenic factors and chemokines are widely expressed in NP tissues when compared with normal nasal mucosa [5, 7–9]. Those two angiogenic factors play a crucial role in angiogenesis [5, 7]. VEGF promotes angiogenesis by increasing capillaries proliferation and migration. Moreover, it increases vascular permeability [5, 7]. It has been postulated that increased VEGF expression in NP tissues might be one of the reasons causing the chronic inflammation and oedema seen in this pathology [7, 14]. Ang-1 is known for its powerful vascular protective effects. These effects are mainly realized by inhibition of vascular inflammation and endothelial cell death [7]. Ang-2's role in angiogenesis is a little more controversial. It has both agonistic and antagonistic effects on this process [7, 15]. Ang-2 contributes the formation of new vessels by increasing the capillary diameter and migration of endothelial cells, especially when VEGF levels increase, when the level of VEGF in the environment decreases, it causes regression in the formation of new vessels [7, 15]. Therefore, it is mostly considered a negative regulatory factor for angiogenesis [7].

Angiogenesis is one important factor for NP pathogenesis. In their study, Karatzanis et al. have found that immune cells VEGF, VEGFR-1, Ang-1, Ang-2, Tie-2A, Tie-2B, SDF-1*α*, and SDF-1*β* mRNA expression to be significantly higher in CRSwNP patients compared to the control group. Similarly, in another study conducted on this issue, an increased vascularity in NP tissues was found in comparison to inferior turbinate tissues [16]. In a recent study conducted by Park et al., authors examined the differences between normal nasal mucosa and nasal polyp tissues on a primary nasal polyp culture. They found that in nasal polyp tissues, vascular endothelial growth factor (VEGF) and Ang-1 levels were significantly increased [7]. In the same study, it was shown that Ang-2 levels were lower in polyp tissues compared to normal nasal mucosa [7]. In another study in which samples from 18 NP patients and 10 control patients were examined, it was shown that the expression of many angiogenic factors including VEGF, VEGFR-1, Ang-1, and Ang-2 was increased in patients with NP and it was emphasized that angiogenic factors play an important role in the pathogenesis of the disease. There are limited number of studies which aim to stop angiogenetic process in NP [5, 7, 10, 17].

Corticotherapy has been used in the treatment of nasal polyposis, especially for its anti-inflammatory effect for many years and accepted as a first-line treatment. In recent years, the antiangiogenic feature of this treatment has also started to be questioned. In a study conducted on primary nasal polyp culture, it was shown that steroids inhibited VEGF expression via the TLR4/Akt/NF-kB pathway [13]. In their in vitro study, Park et al. reported a significant decrease in Ang-1 and VEGF levels, and a significant increase in Ang-2 levels in the dexamethasone-treated group of nasal polyp tissues [8]. In our study, both bevacizumab and propranolol had shown similar effects to dexamethasone on Ang-1, VEGF, and Ang-2 expression levels. We think that the fact that both experimented agents acted similarly as corticosteroid, which is the first line of treatment for NP, is a promising finding for future studies.

Bevacizumab is a FDA approved drug and a monoclonal antibody that inhibits angiogenesis by blocking VEGF-A. It is commonly used in the treatment of metastatic cancers and in the treatment of brain tumors such as glioblastoma multiforme due to its antiangiogenetic properties [14]. In a recent in vitro study, it was shown that bevacizumab inhibited NP proliferation in a dose-depending fashion by decreasing VEGF levels [16].

Bevacizumab has also been used on the nasal mucosa, especially in the treatment of epistaxis due to hereditary hemorrhagic telangiectasia (HHT) [17, 18]. In their study conducted on 32 HHT patients, Karnezis and Davidson [18] reported that submucosal injection or application of bevacizumab as a topical spray to the nasal mucosa reduced the frequency and severity of epistaxis. Similarly, a single dose of 100 mg bevacizumab injection has been reported to be significantly effective over placebo in patients with HHT-related epistaxis [19]. In his study, Guldmann [20] claims that with the administration of 10 cycles of 50 mg intranasal bevacizumab significant improvement was seen in the frequency and severity of epistaxis patients.

Although propranolol is a beta blocker which is mainly used for the treatment of hypertension, atrial fibrillation, and other arrhythmias; it has been shown to decrease VEGF levels in recent years, especially through the P3K/Akt/Enos/VEGF pathway [21, 22]. Due to this antiangiogenic effect, it has become the main drug used in the treatment of hemangiomas [12, 13]. Moreover, like bevacizumab, propranolol has also been used on nasal mucosa to treat epistaxis for its antiangiogenic properties [23–25]. In a study conducted in the pediatric age group, it was reported that there was no statistically significant difference between oral propranolol use and silver nitrate cauterization in terms of recurrent epistaxis control [23]. In another study where the gel form of propranolol was prepared and tested for its efficacy in the treatment of HHT, it was reported that a significant improvement in the severity of epistaxis complaints was achieved by intranasal propranolol administration for 12 weeks [24]. Contis also reported that systemically used propranolol treatment for HHT, decreased the frequency and severity of epistaxis in 11 of 21 patients [25].

Due to the recurrence of nasal polyposis, despite the use of corticotherapy and surgery, which are the main treatment options used today, research is continuing for new treatment possibilities. In recent years, it has been observed that studies on methotrexate have been intensified [5]. *In vitro* study, it was shown that nasal polyposis was performed by reducing proliferation and angiogenesis. In this study, it was noted that VEGF A and Ang-1 levels decreased and Ang-2 levels increased with methotrexate administration [5]. Although research studies for other possible treatment options are focusing on targeted therapies which are, namely, omalizumab, reslizumab, and mepolizubab [5] in the literature, there is only one study investigating the antiangiogenic effects of bevacizumab [22] for NP and no single study about propranolol on this issue.

To investigate the possible efficacy of bevacizumab and propranolol on NP tissues, we chose to test them first on primary nasal polyp culture to advance further steps if successful results are obtained. Our results showed that angiogenesis may play a role in NP development; both Ang-1 and VEGF-A expression are elevated in NP tissues in comparison to normal nasal mucosa tissues and both bevacizumab and propranolol have showed antiangiogenic effects on nasal polyposis tissues. This effect is caused mainly by decreasing VEGF-A and Ang-1 expression and increasing Ang-2 expression in NP tissue which are consistent with the effects of other antiangiogenic treatment options in the literature [5–7]. These antiangiogenic agents may be promising treatment options for the treatment of NP in the future. Further animals and clinical studies are needed to justify these results. Moreover, effects of these drugs on healthy nasal mucosa should also be tested, which we see as a potential weakness of our study.

## 5. Conclusions

Angiogenesis plays an important role in the development of NPS. To conclude, both bevacizumab and propranolol exert an antiangiogenic effect on NP tissues, mainly by decreasing VEGF-A and Ang-1 expression and increasing Ang-2 expression. In the next stage, it is thought that these agents, which are already used in the nose due to their antiangiogenic properties for diseases such as HHT, may become a promising treatment option for recurrent NP cases.

## Figures and Tables

**Table 1:** Expression of VEGF-A, Ang-1, Ang-2 in each group. (−: negative expression, +: Low expression, ++: Moderate expression and +++: High expression).

	VEGF-A	Ang-1	Ang-2
Group 1: Healthy nasal mucosa (control group)	−	+	+++
Group 2: Nasal polyposis group without any treatment	+	++	+
Group 3: Nasal polyposis + propranolol	−	−	++
Group 4 (*n* : 7): Nasal polyposis + bevacizumab	−	−	++

## Data Availability

There are no data loaded as a link. This study does not have a large data to be shared as loaded. The all pathologic data is given as a supplement file in this paper.

## Supplementary Materials

Supplementary Materials The word file includes data of VEGF-A, Angiopoetin 1, Angiopoetin 2 immunohistochemical expression levels of each case of the nasal mucosa and nasal polyp tissues as control, propranolol and bevacizumab applied groups.
