# Supplementary material for: The Effect of Bevacizumab and Propranolol on Nasal Polyposis
Source: Int J Clin Pract. 2022 Oct 12;2022:6174664. doi: 10.1155/2022/6174664 (PMC9581690; doi:10.1155/2022/6174664)
Supplement: Supplementary Materials — The word file includes data of VEGF-A, Angiopoetin 1, Angiopoetin 2 immunohistochemical expression levels of each case of the nasal mucosa and nasal polyp tissues as control, propranolol and bevacizumab applied groups. [file 6174664.f1.docx]

Group VEGFA Angiopoetin1 Angiopoetin 2

| NPolyp control | mild | moderate | mild |
| --- | --- | --- | --- |
| NPolyp control | mild | moderate | mild |
| NPolyp control | mild | high | mild |
| NPolyp control | mild | moderate | mild |
| NPolyp control | mild | moderate | mild |
| NPolyp control | mild | high | mild |
| NPolyp control | mild | moderate | mild |
| Npolyppropranolol | negative | negative | moderate |
| Npolyppropranolol | negative | negative | moderate |
| Npolyppropranolol | negative | mild | moderate |
| Npolyppropranolol | negative | negative | moderate |
| Npolyppropranolol | negative | negative | high |
| Npolyppropranolol | negative | negative | moderate |
| Npolyppropranolol | negative | mild | moderate |
| NPolypbevacizomab | negative | negative | moderate |
| NPolypbevacizomab | negative | negative | moderate |
| NPolypbevacizomab | negative | negative | moderate |
| NPolypbevacizomab | negative | negative | moderate |
| NPolypbevacizomab | negative | negative | moderate |
| NPolypbevacizomab | negative | negative | moderate |
| NPolypbevacizomab | negative | negative | moderate |
| Nmucosa control | negative | mild | high |
| Nmucosa control | negative | mild | high |
| Nmucosa control | negative | mild | high |
| Nmucosa control | negative | mild | high |
| Nmucosa control | negative | mild | high |
| Nmucosa control | negative | mild | high |
| Nmucosa control | negative | mild | moderate |
| Nmucosa propronalol | mild | mild | moderate |
| Nmucosa propronalol | mild | negative | mild |
| Nmucosa propronalol | mild | negative | mild |
| Nmucosa propronalol | mild | negative | mild |
| Nmucosa propronalol | mild | negative | mild |
| Nmucosa propronalol | mild | negative | moderate |
| Nmucosa propronalol | mild | negative | mild |
| NMucosabevacizumab | mild | negative | moderate |
| NMucosabevacizumab | mild | negative | moderate |
| NMucosabevacizumab | mild | negative | moderate |
| NMucosabevacizumab | mild | negative | moderate |
| NMucosabevacizumab | mild | negative | moderate |
| NMucosabevacizumab | mild | negative | moderate |
| NMucosabevacizumab | mild | negative | moderate |
